# Cytologically indeterminate thyroid nodules: increased diagnostic performance with combination of US TI-RADS and a new scoring system

**DOI:** 10.1038/s41598-017-07353-y

**Published:** 2017-07-31

**Authors:** Ya-Ping He, Hui-Xiong Xu, Chong-Ke Zhao, Li-Ping Sun, Xiao-Long Li, Wen-Wen Yue, Le-Hang Guo, Dan Wang, Wei-Wei Ren, Qiao Wang, Shen Qu

**Affiliations:** 1 Department of Medical Ultrasound, Shanghai Tenth People’s Hospital, Ultrasound Research and Education Institute, Tongji University School of Medicine, Shanghai, 200072 China; 2 Thyroid Institute, Tongji University School of Medicine, Shanghai, 200072 China; 3 Shanghai Center for Thyroid Diseases, Shanghai, 200072 China; 4 Department of Endocrinology & Metabolism, Shanghai Tenth People’s Hospital, Tongji University School of Medicine, Shanghai, 200072 China

## Abstract

To investigate the diagnostic performance of combination of ultrasound (US) thyroid imaging reporting and data system (TI-RADS) and a new US scoring system for diagnosing thyroid nodules (TNs) with indeterminate results (Bethesda categories III, IV and V) on fine-needle aspiration (FNA) cytology. 453 patients with 453 cytologically indeterminate TNs were included in this study. Multivariate analyses were performed to construct the scoring system. The diagnostic performances of TI-RADS and the combined method were evaluated and compared. Multivariate analyses revealed that marked hypoechogenicity, taller than wide shape and absence of halo sign were independent predictors for malignancy in cytologically indeterminate TNs. Scoring system was thereafter defined as follows: risk score (RS) = 3.2 x (if marked hypoechogenicity) + 2.8 x (if taller than wide shape) + 1.3 x (if absence of halo sign). Compared with TI-RADS alone, the areas under the receiver operating characteristic curves (AUC), specificity, accuracy and positive predictive value (PPV) of the combined method increased significantly with 0.731 versus 0.569, 48.5% versus 14.1%, 76.2% versus 62.3%, and 70.9% versus 59.9%, respectively (all *P* < 0.05). The combination of TI-RADS and new US scoring system showed superior diagnostic performances in predicting malignant TNs with indeterminate FNA cytology results in comparison with TI-RADS alone.

## Introduction

Ultrasound (US) guided fine-needle aspiration (FNA) has been regarded as the most cost-effective method in differentiating malignant from benign thyroid nodules (TNs) with high sensitivity and specificity, which significantly decreases the number of unnecessary surgery for benign nodules^1–3^. According to the Bethesda System for Reporting Thyroid Cytopathology, the FNA results are divided into six categories to unify the terminology among laboratories and pathologists as following: (i) nondiagnostic/unsatisfactory; (ii) benign; (iii) atypia of undetermined significance/follicular lesion of undetermined significance (AUS/FLUS); (iv) follicular neoplasm/suspicious for follicular neoplasm (FN); (v) suspicious for malignancy (SUSP), and (vi) malignant^4^. Among these categories, the indeterminate samples (i.e. Bethesda categories III, IV and V) account for approximately 10–30%, for which repeating FNA or surgery is usually recommended^5^. The uncertain malignancy risk of indeterminate samples leads to uncertainty for choosing treatment in clinical practice^6^. Some studies reported that repeat FNA can get a definitive cytologic diagnosis in Bethesda category III TNs; however, there are 10–30% nodules remaining the same cytologic results as the initial diagnosis, arguing against the role of repeat FNA^7–10^. For those TNs with final benign results in surgical pathology, resection approach would be regarded as unnecessary^11^. Although molecular marker tests are proven to be promising in diagnosing TNs in many studies, the high price and non-availability in all hospitals make them not suitable to be used as basic examination in the clinical application^12–14^.

There is general agreement that conventional ultrasound (US) plays an essential role in detecting malignant TNs and in selecting TNs for FNA. These roles are primarily attributed to the ability of US in evaluating malignancy risk in TNs^15^. Thyroid Imaging Reporting and Data System (TI-RADS) is aimed to standardize the correlation between US features and malignancy risk, which achieves better communication between radiologists and clinicians. Horvath *et al*.^16^ firstly proposed TI-RADS using ten malignancy associated US features to stratify the malignant risk in each category. Subsequently, another version of TI-RADS using twelve US features of TNs was reported by Park *et al*.^17^. Nonetheless, both systems appeared difficult to be applied in routine clinical practice owing to their complexity for interpretation^16–18^.

Recently, another version of TI-RADS designed by Kwak *et al*. adopted the number of suspicious US characters such as solid component, hypoechogenicity or marked hypoechogenicity, irregular margins, microcalicifications or mixed calcifications, and taller-than-wide shape to stratify malignancy risk of TNs, which can be relatively easy to be used in clinical practice^18, 19^. In this system, TI-RADS categories 3, 4a, 4b, 4c, and 5 are defined by no, one, two, three or four, and five suspicious US features, respectively. The risk of malignant in TI-RADS categories 3, 4a, 4b, 4c and 5 are 2–2.8%, 3.6–12.7%, 6.8–37.8%, 21.0–91.9%, and 88.7–97.9%. Several investigators had applied this TI-RADS category in assessing the malignancy risk for TNs, which proved that this TI-RADS category could efficiently predict malignant TNs^5, 20–22^. In addition, Ko *et al*. had investigated the diagnostic performances of this TI-RADS in differentiating malignant from benign TNs, with 99.1% sensitivity, 35.9% specificity, 52.5% accuracy, 35.5% positive predictive value (PPV) and 99.1% negative predictive value (NPV), respectively^23^. However, the high sensitivity and NPV obtained by Ko *et al*. were based on FNA cytology results in which the cytologically indeterminate TNs were excluded. To date, the knowledge about the diagnostic efficiency of TI-RADS on cytologically indeterminate TNs is still limited. Moreover, some other US signs, such as halo sign, nodule size and vascularity, which were not considered by Ko *et al*.^23^, were proposed as significant predictors in diagnosing malignant TNs in other previous studies^24–26^. Therefore, these US features should be considered when developing a scoring system to predict malignancy in indeterminate nodules. Furthermore, the specificity (25.6–35.9%) of Kwak’s TI-RADS was relatively low in diagnosis of malignant nodules, thus further refinement of diagnostic methods is needed^23, 27, 28^.

In the current study, the primary aim was to assess the diagnostic performance of Kwak’s TI-RADS for TNs with indeterminate FNA cytology results. In addition, a new scoring system based on the possible US predictors for malignancy in indeterminate TNs was proposed. The second aim was to evaluate whether the scoring system or the combination of TI-RADS and the scoring system would increase the diagnostic performance in comparison with TI-RADS alone.

## Materials and Methods

This retrospective study was approved by the Ethical Committee of the Shanghai Tenth People’s Hospital of Tongji University School of Medicine and the requirement to obtain informed consent was waived. This study performed in accordance with the relevant regulations and guidelines.

### Study population

We retrospectively reviewed our institutional database for the TNs with US-guided FNA cytology results during the period of March 2013 to September 2016. FNA was conducted for a total of 4850 consecutive patients with 4967 TNs during this period. The TNs were subject to FNA due to one or several suspicious US features according to Kwak’s TI-RADS, the requirement of patients, or referral from the clinicians. Patients underwent FNA for single TN in 4754 (98.0%) patients, for two nodules in 75 (1.5%) patients, and for three nodules in 21 (0.4%) patients. For patients with multiple nodules who had undergone FNA, the most suspicious one was scheduled for analysis; otherwise the largest one was selected. TNs with the following features were included: (a) nodules with indeterminate cytology results (i.e. Bethesda categories III, IV and V); (b) nodules on which thyroidectomy had been performed; (c) nodules were all larger than 5 mm in size measuring. 456 TNs in 456 patients met the inclusion criteria and 3 TNs were excluded for the reason of losing US images. Finally, 453 nodules in 453 patients with indeterminate cytology results were analyzed in this study and all of them were finally pathologically confirmed after surgery.

### US and US-guided FNA cytology

US examinations were performed using Logiq E9 (GE Medical Systems, Milwaukee, WI, USA; 6–15 MHz linear transducer), Siemens S2000 (Siemens Medical Solutions, Mountain View, CA, USA; 5–14 MHz linear transducer), or IU22 (Philips Medical Systems, Bothell, WA, USA; 5–12 MHz linear transducer) instruments by one of five radiologists with more than 3 years of experience in thyroid imaging. All patients underwent US examination in a supine position with slightly dorsal flexing of head. US images were obtained both on transverse and longitudinal axis for each target nodule and nodules size were defined by the maximum diameter at US images. After that, the optimal image features of the target nodule were obtained by adjusting the machine settings. All the US images were recorded and stored for further analysis.

US-guided FNA was performed using a 22-gauge PTC needle attached to a 5-mL disposable plastic syringe with freehand technique. The process of aspiration was conducted at least twice for each target lesion. Then, the materials aspirated from the target lesion were expelled onto glass slides and smeared, which were placed immediately in 95% alcohol for the following hematoxylin-eosin staining. Cytopathologists were not on site during the aspiration procedure, and cytology results were interpreted on the basis of Bethesda system.

### Image interpretation

The US characteristics of TNs were retrospectively interpreted by two independent radiologists who did not participate in acquiring US images, and both of them were blind to the final cytology results (one radiologist with 5 years’ experience in thyroid US and the other with 3 years’ experience). When discordance appeared between the two investigators, the final decision was made by another senior radiologist with more than 10 years of experience in thyroid US image. All the target TNs were assessed on gray-scale US and color Doppler US with the following features: internal composition, echogenicity, margin, calcifications, shape, halo sign, nodule size, and internal vascularity. Internal composition was classified as solid or cystic portion ≤50% and cystic portion >50%, according to the ratio of cystic component to solid component. Echogenicity was interpreted as hyper-, iso-, or hypo-echogenicity in comparison of normal thyroid gland, while marked hypoechogenicity was defined if the echogenicity was relatively hypoechoic in comparison of surrounding strap muscle. Margin was classified as well or poorly circumscribed. Calcifications, if present, were defined as macrocalcificaions for which the calcifications were >1 mm in diameter, or microcacifications for which the calcifications were visualized as tiny punctuate hyperechonic foci of ≤1 mm in diameter with or without acoustic shadows. When both macrocalcification and microcalcificaion appeared in the same nodule, we classified it as microcalcificaion. Shape was classified as taller than wide when the anteroposterior dimension of the target nodule was greater than its transverse dimension, or else defined as wider than tall. Halo sign was interpreted as a hypo-echoic rim surrounding the lesion^25^. Nodule size was defined as the maximum diameter at US images. Vascularity was defined as no internal and peripheral blood flow (type I), or predominant pattern of peripheral blood flow (type II), predominant pattern of internal blood flow (type III)^24^.

There were three settings in diagnosis of malignant TNs in this study. Setting 1: TI-RADS method alone; setting 2: scoring system alone, the eight US features mentioned above were adopted to construct the scoring system to obtain a cut-off point in predicting malignancy; setting 3: a combination of TI-RADS and the new scoring system.

### Statistical analysis

All the statistical analyses in this study were performed using the SPSS 20.0 software (SPSS, Chicago, IL) and MedCalc software (Mariakerke, Belgium). Two-tailed *P* values < 0.05 were considered statistically significant.

Mean values ± standard deviations (SD) were used for continuous data with normal distribution while counts and percentages were used for categorical data. An independent-samples *t* test was performed to test the differences of patient age and nodule size between benign and malignant TNs. A χ^2^ test was performed to compare the differences of patient sex and US features between benign and malignant TNs. For statistical analysis, TNs of TI-RADS category 3 were considered as benign, whereas TI-RADS category 4 or 5 were considered as malignant. To evaluate the relationship between US features and malignant nodules, multivariate logistic regression analysis was used. After that, the odds ratios (ORs) with relative 95% confidence intervals (CIs) and regression coefficient (β) of statistically significant US predictors were obtained according to multivariate analysis. The risk score (RS) in each statistically significant US feature was multiplied by the β value, and then the scoring system was constructed using the sum of RS in all US predictors. Based on the scoring system, the score of malignancy can be obtained at each nodule.

Afterwards, by using receiver operating characteristic (ROC) curves, the optimal cut-off value of scoring system can be calculated when the Youden index (i.e. YI = sensitivity + specificity-1) was maximum. In the combined method, if the RS of a nodule was less than the cut-off value, we would degrade the TI-RADS category, such as from TI-RADS 4a to 3, 4b to 4a, 4c to 4b, and 5 to 4c; otherwise, we would upgrade the TI-RADS category, such as from TI-RADS 4c to 5, 4b to 4c, 4a to 4b, and 3 to 4a. To assess the diagnostic performances of the three settings in diagnosing malignant TNs, ROC curves was evaluated to obtain the areas under the ROC curves (AUC), sensitivity, specificity, accuracy, PPV and NPV values. The comparisons of AUCs were performed using Z score test. McNemar test was performed to compare the differences in sensitivity, specificity and accuracy, while chi-square test or Fisher exact test was performed in PPV and NPV.

## Results

### Basic characteristics

**Figure 1:**
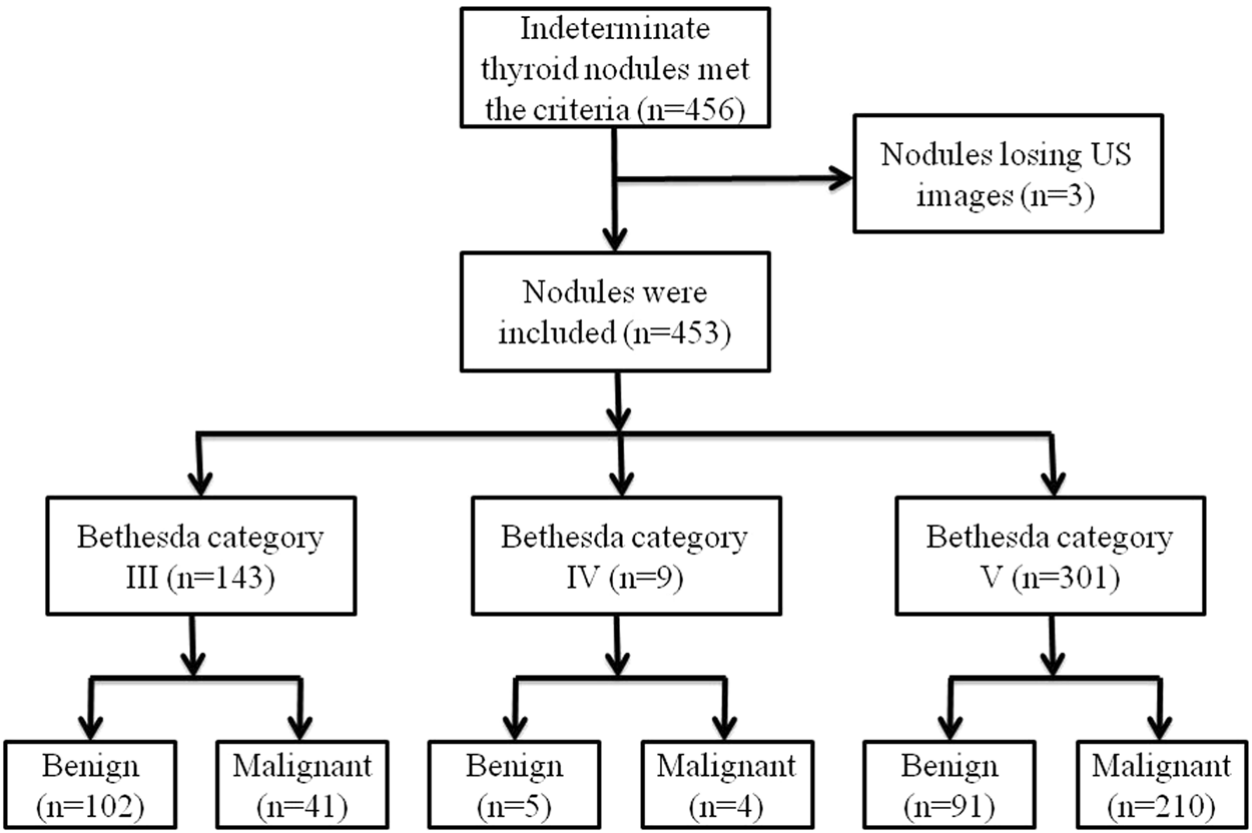
Flowchart of selected patients with thyroid nodules.

Of the 453 TNs with indeterminate FNA results, 198 (43.7%) were benign and 255 (56.3%) were malignant. The pathological findings of the 453 TNs are shown in Table 1. Among the TNs, there were 143 Bethesda category III nodules in which 41 (28.7%) were malignant, 9 Bethesda category IV nodules in which 4 (44.4%) were malignant, and 301 Bethesda category V nodules in which 210 (69.8%) were malignant (Fig. 1). Benign TNs were significantly larger than malignant TNs in size (mean size, 13.4 mm ± 8.4 versus 10.2 mm ± 5.8; *P* < 0.001). Patients with benign TNs were older than those with malignant TNs (mean age, 54.9 years ± 11.6 versus 48.3 years ± 13.9, respectively; *P* < 0.001). There was no statistically significant association between malignant TNs and sex. When comparing the differences of conventional US features between malignant and benign TNs, composition, echogenicity, margin, calcifications, shape, halo sign, vascularity showed significant associations with risk of malignancy with all *P* < 0.001 (Table 2).

**Table 1:** Histopathologic findings in the 453 thyroid nodules treated with surgery.

Findings	No. of nodules
Malignant	255
Papillary carcinoma	248
Medullary carcinoma	4
Follicular carcinoma	2
Poorly differentiated carcinoma	1
Benign	198
Nodular goiter	106
Hashimoto’s nodule	53
Subacute thyroiditis	3
Adenomatous goiter	19
Follicular adenoma	17

**Table 2:** Basic demographic characteristics and conventional US features in diagnosing all thyroid nodules according to malignant and benignity.

Parameter	All nodules (n = 453)	Malignant (n = 255)	Benign (n = 198)	*P* value
Patient				
Mean age (y)*	…	48.3 ± 13.9 (10–82)	54.9 ± 11.6 (23–75)	<0.001
Sex				0.527
Female	363	207 (57.0)	156 (43.0)	
Male	90	48 (53.3)	42 (46.7)	
Nodule				
Mean size (mm)*	…	10.2 ± 5.8 (5.0–36.0)	13.4 ± 8.4 (5.0–42.0)	<0.001
Composition				<0.001
Cystic portion > 50%	6	0 (0)	6 (100)	
Cystic portion ≤ 50%	71	17 (23.9)	54 (76.1)	
Solid	376	238 (63.3)	138 (36.7)	
Echogenicity				<0.001
Hyperechogenicity	6	1 (16.7)	5 (83.3)	
Isoechogenicity	95	11 (11.6)	84 (88.4)	
Hypoechogenicity	270	168 (62.2)	102 (37.8)	
Marked hypoechogenicity	82	75 (91.5)	7 (8.5)	
Margin				<0.001
Well circumscribed	300	125 (41.7)	175 (58.3)	
Poorly circumscribed	153	130 (85.0)	23 (15.0)	
Calcifications				<0.001
No calcifications	211	78 (37.0)	133 (63.0)	
Macrocalcifications	46	10 (21.7)	36 (78.3)	
Microcalcifications	196	167 (85.2)	29 (14.8)	
Shape				<0.001
Wider than tall	251	68 (27.1)	183 (72.9)	
Taller than wide	202	187 (92.6)	15 (7.4)	
Halo sign				<0.001
Present	86	16 (18.6)	70 (81.4)	
Absent	367	239 (65.1)	128 (34.9)	
Vascularity				<0.001
None	131	85 (64.9)	46 (35.1)	
Type II	188	82 (43.6)	106 (56.4)	
Type III	134	88 (65.7)	46 (34.3)	

*Indicates means ± standard deviations. Data are ranges, otherwise are percentages in the parentheses.

Type II = predominant pattern of peripheral blood flow; Type III = predominant pattern of internal blood flow.

### Multivariate logistic regression analysis

**Figure 2:**
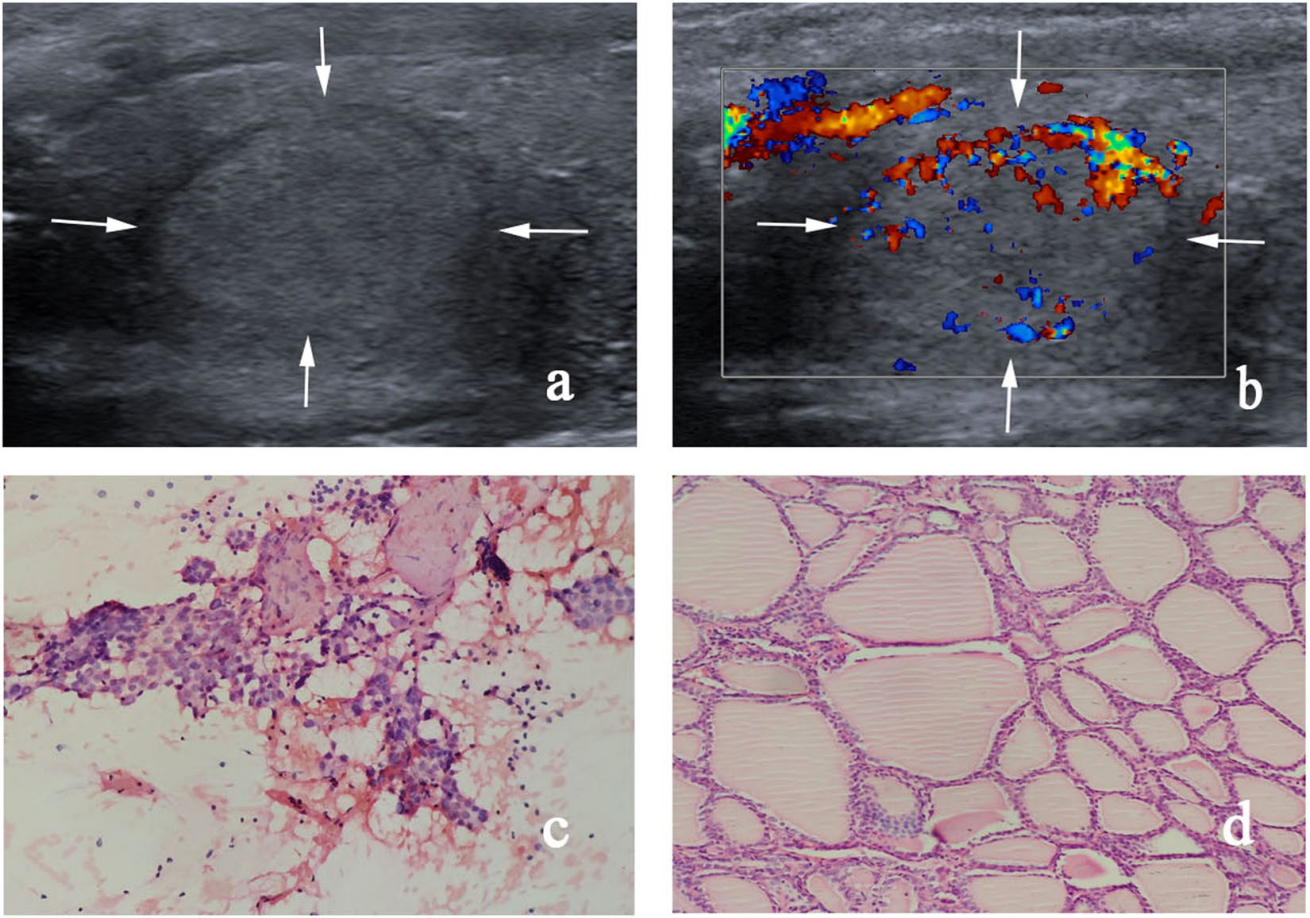
Adenomatous goiter is surgically confirmed for a 44-year-old woman. (**a**) Gray-scale US shows solid, isoechoic, well circumscribed, no calcifications, wider than tall, and halo sign in the nodule. (**b**) Color Doppler US shows type II vascularity in the nodule. Category 4a is classified based on TI-RADS alone, while category 3 is finally classified using the combined method (RS = −1.3). (**c**) This thyroid nodule is cytologically confirmed to be Bethesda category III by FNA (hematoxylin-eosin stain; original magnification, ×200). (**d**) This thyroid nodule is surgically confirmed to be an adenomatous goiter by histological specimen (hematoxylin-eosin stain; original magnification, ×200).

Multivariate logistic regression analysis including all the significant US features in this study was conducted and showed that marked hypoechogenicity (OR = 38.997; 95% CI: 1.777–855.954; *P* = 0.020), taller than wide shape (OR = 18.520; 95% CI: 8.221–41.721; *P* < 0.001) and absence of halo sign (OR = 2.912; 95% CI: 1.170–7.248; *P* = 0.022) were independent risk factors in predicting malignant TNs. Nodule size (OR = 0.940; 95% CI: 0.890–0.991; *P* = 0.022) was significantly associated with low risk in differentiating malignant TNs. A final predicting model of scoring system was constructed on the basis of 3 independent risk factors obtained from multivariate logistic regression analysis (Table 3). RS of scoring system was defined as follows: RS = 3.7 x (if marked hypoechogenicity) + 2.9 x (if taller than wide shape) + 1.1 x (if absence of halo sign). Each nodule can obtain a RS according to the formula above. By using ROC curve, the most optimal cut-off point of RS for scoring system in predicting malignant from benign TNs was 2.0. With this cut-off point, TI-RADS categories were degraded and upgraded in 205 (45.3%, 205/453) and 151 (33.3%, 151/453) TNs respectively, leading to a change in TI-RADS category in 78.6% of all TNs. There was a case which was degraded from TI-RADS category 4a to 3 shown in Fig. 2. For nodules with TI-RADS category 3 and RS less than 2.1 (6.4%, 29/453) or TI-RADS category 5 and RS more than 2.1(15.0%, 68/453), the TI-RADS category was not changed.

**Table 3:** Risk score of independent conventional US parameters in predicting malignant thyroid nodules according to multivariate logistic regression.

Parameter	β	SE	OR (95%CI)	*P* value	RS
Marked Hypoechogenicity	3.663	1.576	38.997 (1.777, 885.954)	0.020	3.7
Taller than wide shape	2.919	0.414	18.520 (8.221, 41.721)	<0.001	2.9
Absence of halo sign	1.069	0.465	2.912 (1.170, 7.248)	0.022	1.1

β = regression coefficient; SE = standard error; OR = odds ratios; CI = confidence interval; RS = risk score.

### Diagnostic performances

**Figure 3:**
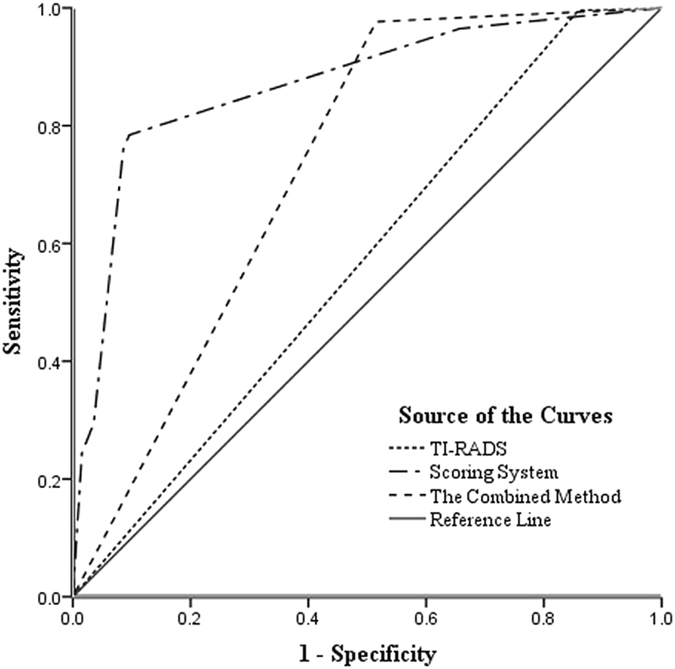
Receiver operating characteristic (ROC) curves of the three settings.

Using TI-RADS category 4a and RS 2.0 in scoring system as the cut-off point for predicting malignancy, the diagnostic performances of the three settings were shown in Table 4 (Fig. 3). Among these performances, the AUC, specificity, accuracy and PPV of the combined method increased significantly in comparison of TI-RADS alone, with no significant decrease in sensitivity and NPV. In addition, we also compared the diagnostic performances between scoring system and the combined method, and found the specificity significantly increased (90.4% versus 48.5%) whereas the sensitivity significantly decreased (78.4% versus 97.6%) for the scoring system alone in comparison with the combined method (all *P* < 0.001).

**Table 4:** Diagnostic performances of the three methods in the diagnosis of thyroid nodules.

Parameter	Sensitivity	Specificity	Accuracy	PPV	NPV
Setting 1	99.6	14.1	62.3	59.9	96.6
Setting 2	78.4	90.4	83.7	95.7	76.5
Setting 3	97.6	48.5	76.2	70.9	94.1

Setting 1 = TI-RADS; Setting 2 = scoring system; Setting 3 = the combined method of TI-RADS and scoring system.

## Discussion

Management of TNs with cytologically indeterminate FNA results is still challenging on account of the difficulty in diagnosing cytology morphology. Up to now, several attempts have been made to assess the diagnostic performances for cytologically indeterminate TNs. The TI-RADS classification which is based on using suspicious US features to stratify malignancy of TNs had been developed as various versions, in which the version of Kwak *et al*. was the easiest method in the clinical application and was used in our study. The high sensitivity and NPV (99.6% and 96.6%) obtained in the current study were consistent with the previous studies (sensitivity, 97.4–99.1% and NPV, 98.1–99.1%)^23, 27, 28^. In addition, a recent report used the TI-RADS proposed by Horvath *et al*. to differentiate malignant from benign TNs with indeterminate cytology results, which achieved 67% sensitivity and 19% specificity^26^. Compared with our results, the 67% sensitivity was quite lower than 99.6% sensitivity diagnosed by the TI-RADS of Kwak *et al*. in the current study. However, a common problem of relatively low specificity existed either in the current study or in the previous studies should be noticed (range, 14.1–35.9%).

The TI-RADS proposed by Kwak *et al*. was on account of the probability of malignancy in various US features. Then, the TI-RADS category was constructed by a risk-stratifying model including five statistically significant US predictors obtained from logistic regression analysis. Nevertheless, with different cohorts, especially for TNs with indeterminate FNA results in the current study, the probability of malignancy of the risk factors might be different from the TI-RADS of Kwak *et al*. In addition, except for the five US predictors in TI-RADS, other US features such as halo sign, nodule size and vascularity had previously been demonstrated the promising capacity to predict malignant from benign TNs^25, 26, 29–31^. Therefore, on the basis of TI-RADS, we hypothesized combining TI-RADS and scoring system obtained from logistic regression analysis can improve the specificity of TI-RADS alone. Eight US features mentioned above were subject to logistic regression analysis, then the scoring system comprised by three independent risk factors of US predictors (i.e. marked hypoechogenicity, taller than wide shape, and absence of halo sign) assigned with individual risk score was established. Among the three independent risk factors in the current scoring system, hypoechogenicity and taller than wide shape were also present in the TI-RADS of Kwak *et al*., the potential overlap of the two US features between the two methods indicated the two parameters played an important role in predicting malignant TNs. However, for the scoring system in the present study, the US features of solid component, poorly circumscribed margin and microcalcificaion were all not the independent predictors compared with TI-RADS from Kwak *et al*., which might be owing to only indeterminate TNs were included in our study. In addition, nodule size in the present study seemed to be the predictors of low-risk thyroid carcinoma, as found in previous study that malignant TNs were smaller than benign ones in sizing measuring^32^, which was conversely reported in another study^26^. The different results for nodule size in predicting malignant TNs might be caused by the discrepancy of patients enrolled. When combining TI-RADS with the cut-off value of 2.0 in the scoring system, the diagnostic performances statistically improved in AUC, specificity, accuracy and PPV values, with no statistically decreasing in sensitivity and NPV compared with TI-RADS alone, which indicated there would be approximately three times patients with indeterminate cytology results can be degraded from 4a to 3 category, for whom follow-up can be recommended in lieu of thyroidectomy, compared with the initial diagnosis of TI-RADS alone. Additionally, compared with the combined method, the specificity significantly increased and the sensitivity significantly decreased for scoring system. In order to remain the high sensitivity of TI-RADS, and improve the specificity simultaneously, therefore, we recommend the combined method other than scoring system alone.

In a recent study in which Yoon *et al*. compared the diagnostic performances of six guidelines published in managing TNs with a large sample of 4696 TNs in 4585 patients^27^. Among the six guidelines, there were three TI-RADS criteria versions derived from Russ *et al*.^33^, Kim *et al*.^34^, and Kwak *et al*., in which the sensitivity and specificity in diagnosing malignant TNs were 95.2% and 52%, 87% and 83.1%, and 98.8% and 25.6%, respectively. For the combined method in the present study, the sensitivity were the highest in comparison of the three methods mentioned above; meanwhile, the specificity were higher than that of TI-RADS from Kwak *et al*. above^27^.

In addition, another study group also evaluated whether the TI-RADS from Kwak *et al*. can be efficiently performed to diagnose malignant TNs, with the results of sensitivity 97.4% and specificity 29.3%, which were all lower than that of the combined method in our study^28^. Higher specificity is quite essential in the management of TNs, which can decrease the false-positive patients, so as to reduce the rates of unnecessary surgery. Therefore, on account of TI-RADS, using the cut-off value of scoring system to upgrade or degrade the TI-RADS category might be a promising method in predicting malignant TNs with indeterminate FNA cytology results.

There were several limitations in the current study. First, the sample of 453 nodules in this study was relatively small and a large scale specimen was needed for further studies. Second, because of the retrospective design in this study, and selection bias may have existed in patient enrollment. Of the 453 nodules with indeterminate cytology results in our study, a majority of these nodules were selected for having suspicious US features, which might not represent the prevalence rate of nodules with indeterminate cytology results in the general population. However, with indeterminate cytology results, the probability of malignancy of the US risk factors of TNs might be different from those common TNs from general population. Therefore, our study had evaluated the diagnostic efficiency of US features in the TNs with indeterminate cytology results and obtained good diagnostic performance in predicting malignant TNs. Third, we didn’t consider the possible coexistence of thyroid autoimmunity, a condition frequently resulting in a higher rate of indeterminate or suspicious cytological results^35^, which can be regarded as a research direction in the future study. In addition, the final diagnosis was all pathologically confirmed with surgery, which might inevitably lead to the high malignancy rate of TNs. This factor might account for the high sensitivity in our study. Last, the present study was a single-site and retrospective study, so the results obtained in our study need to be verified by prospective multicenter study in the future.

To conclude, with its higher specificity and accuracy compared with TI-RADS alone, the combined method of TI-RADS and the new scoring system may be an effective way to be applied in the management of TNs with indeterminate FNA cytology results. In addition, future prospective and large scale studies are needed to validate the results.
